# Incidence and treatment of femur fractures in adults with osteogenesis imperfecta: an analysis of an expert clinic of 216 patients

**DOI:** 10.1007/s00068-018-1005-9

**Published:** 2018-09-22

**Authors:** Wouter Alexander Goudriaan, Gerrit Jan Harsevoort, Marije van Leeuwen, Antonius Adrianus Franken, Guus Johannes Maria Janus

**Affiliations:** 1 Department of Orthopaedic Surgery, Isala, Zwolle, The Netherlands; 2 Department of Internal Medicine, Isala, Zwolle, The Netherlands

**Keywords:** Osteogenesis imperfecta, Femur fracture, Non-union, Adult

## Abstract

**Purpose:**

Osteogenesis imperfecta (OI) is characterized by increased bone fragility and susceptibility for fractures. A few studies described and compared treatment modalities for femur fractures in children with OI. However, no cohort studies on adults with OI have been published. This study on adult OI patients aims to give insight into the incidence of femur fractures and non-unions and its best treatment options to avert non-union.

**Methods:**

In this retrospective, descriptive study of the OI expert clinic in The Netherlands, all medical charts of patients 16 years or older were analyzed for femur fracture incidence, non-union rate and treatment modality.

**Results:**

Of 216 OI patients, 34 patients suffered a femur fracture with 12 patients having more than 1 femur fracture. For all types of femur fractures, the incidence was 651 fractures per 100,000 person-years annually. In 49 total fractures, 10 fractures resulted in a non-union, mostly shaft fractures of type 4 OI patients. Surgically treated shaft fractures had the best outcomes for non-union.

**Conclusions:**

OI adults were prone to developing femur fractures and non-unions. Especially type 4 OI adults, with conservatively treated shaft fractures, were at high risk for non-unions.

## Introduction

Osteogenesis imperfecta (OI) is the common name for a heterogeneous group of connective tissue disorders primarily characterized by increased bone fragility, also known as brittle bone disease. 90% of patients with OI have mutations in COL1A1 or COL1A2 gene, which, respectively, encodes for alpha-1 and alpha-2 chains in type 1 collagen [1]. Because bone tissue is mainly composed of type 1 collagen, this disorder is associated with increased risk of fractures and skeletal deformation [2]. Besides the increased incidence of fractures, there is a variable occurrence of blue sclerae, dentinogenesis imperfecta, hyperlaxity, hearing loss and short stature [3].

This clinical variability and pattern of inheritance has led to the Sillence classification of four OI types (types 1–4) [4]. Additional types have been added later; thereof type 5 is worldwide accepted. Type 1 represents the most common and mildest form of OI, characterized by blue sclerae and frequent presence of dentinogenesis imperfecta. An increased fracture risk exists, without serious deformity of bones. Type 2 is a perinatal lethal form. Type 3 is the most severe (non-lethal) form of OI with multiple fractures, progressive deformity and short stature, often wheelchair bound. Type 4 shows, in addition to increased fracture risk, a variable degree of deformity with normal sclerae. Type 5 is characterized by mild to severe weak bones and progressive calcifications of the interosseous membrane of forearm and lower leg. This type often results in hyperplastic callus formation after fractures [3]. Bone fragility, and thereby fracture risk, increases in the order Type 1 < Types 4, 5 < Type 3 < Type 2 [5].

Although femur fractures are common in OI patients, no literature specifies fracture and non-union incidence in adults, most likely explained by the rarity of this disease [6–8]. Non-union rate is expected to be higher in OI adults than non-OI adults, as surgical treatment is more challenging due to anatomical and bone-related abnormalities [7, 9, 10].

The aim of this descriptive study is to give an overview on the incidence of femur fractures and non-unions in osteogenesis imperfecta adults and to review our experience giving the best possible advices on treatment options with non-union as outcome measure.

## Patients and methods

### Study population

This retrospective study is performed in the OI expert clinic for adults in Zwolle, The Netherlands. Medical records of all OI patients were retrieved and patients with a femur fracture at age 16 years or older until end of 2015 were included. Patients with an osseous primary tumor, metastatic disease or prednisone use were excluded due to an increased fracture risk. At time of multidisciplinary intake and screening, radiographs were taken in case of recent bone fracture or localized pain, suggesting a possible fracture. Patients with a possible history of femur fracture, but with missing medical records, were contacted and asked for permission of obtaining documentation from other hospitals or general practitioners.

Besides demographic characteristics, we recorded type of OI proposed by Sillence and the additional type 5. Femur fractures were subdivided in categorical variables according to the AO/OTA Fracture and Dislocation Classification of femur fractures (proximal, shaft, distal), type of treatment [intramedullary nailing (IN), plate fixation (PF), conservative] and outcome (union, non-union). Radiographic follow-up was used to determine union. Union was defined as the presence of bridging callus in at least 3 of 4 cortices, evaluated on radiographs in two transverse levels [11]. Non-union was defined as non-radiographic changes to union or the absence of bridging callus of two or more cortices, evaluated on radiographs in two transverse levels, for at least 6 months after surgical or conservative treatment [12, 13].

### Statistical methods

As this study contains relatively small number of subjects, a descriptive study was performed. Descriptive statistics were used to analyze the results using StataCorp. 2013 (Stata Statistical Software: Release 13. College Station, TX: StataCorp LP). Categorical variables were expressed as percentage and metric variables as mean and standard deviation.

### Ethics

No medical ethical approval was required after assessing this study protocol by the local Medical Ethical Committee. All patients provided written consent for obtaining medical records.

## Results

**Fig. 1:**
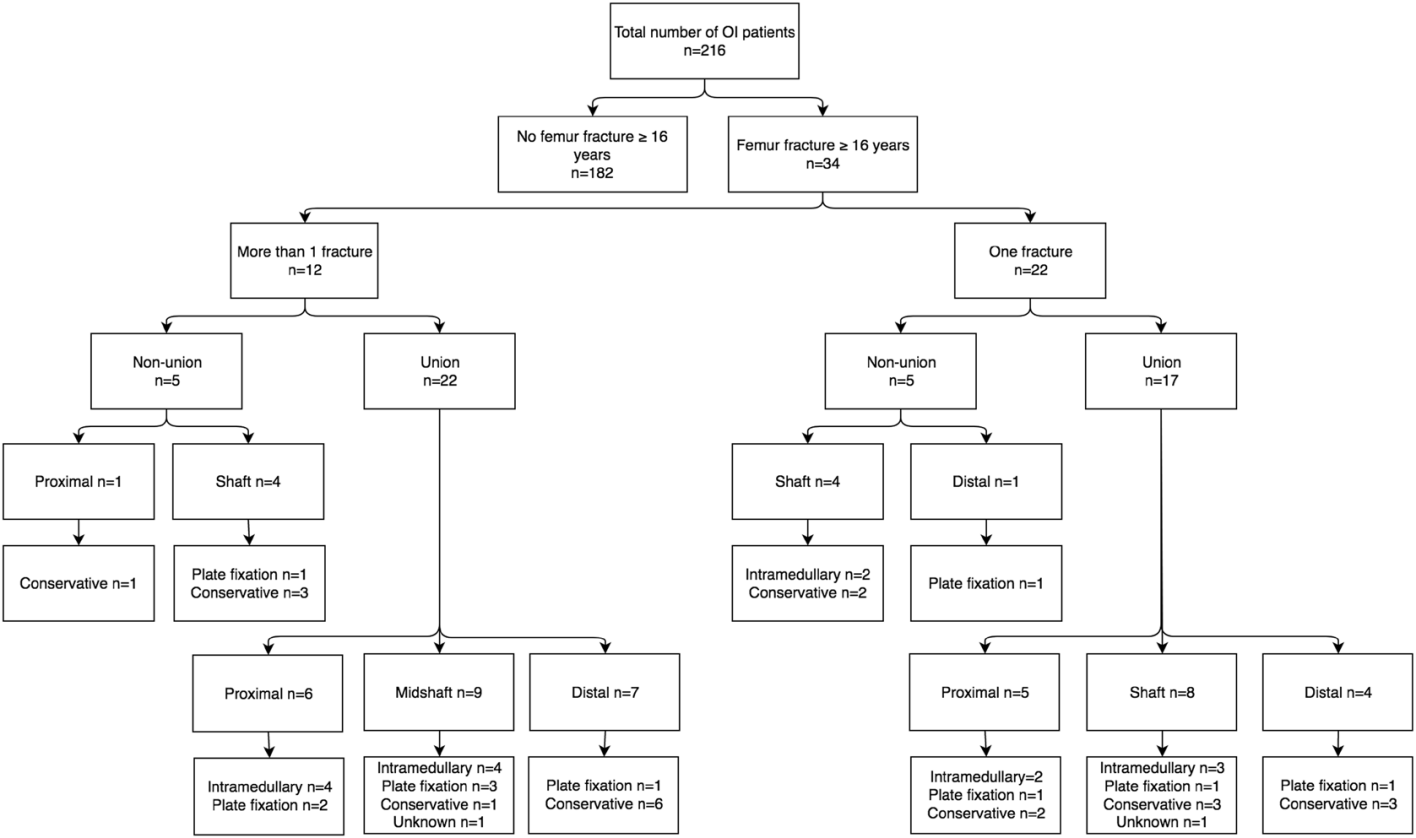
Flow diagram of fracture pattern and treatment

Until the end of 2015, 216 patients were admitted to the orthopaedic expert clinic. No patient was excluded by exclusion criteria. Demographic characteristics of study subjects with OI were given in Table 1. Subdivision of patients was based on history of femur fracture and, subsequently, history of non-union. 34 (15.2%) patients suffered a femur fracture (Fig. 1). 12 of these patients suffered more than one femur fracture (27 fractures in total), resulting in a total of 49 femur fractures (Fig. 1).

**Table 1:** Demographic characteristics of study subjects with osteogenesis imperfecta

	All patients (*n* = 216)	No femur fracture (*n* = 182)	Femur fracture (*n* = 34)
Age (years)	41.0 ± 15.3	40.4 ± 15.4	44.1 ± 14.7
Gender (male, %)	75 (34.7)	59 (32.4)	16 (47.1)
OI type (*n*, %)			
1	152 (70.4)	137 (75.3)	15 (44.1)
2	0 (0)		
3	29 (13.4)	20 (11)	9 (26.5)
4	33 (15.3)	24 (13.2)	9 (26.5)
5	2 (0.9)	1 (0.6)	1 (2.9)

Data are presented as mean ± standard deviation or number of patients and percentages of group

### Influence of OI type on healing rate

OI type 3 and 4 patients were prone to femur fractures; respectively, nine (31%) and nine (27.3%) of these OI patients had at least one fracture. Of all type 1 patients, 15 (9.9%) suffered at least one femur fracture. Of a total of 49 femur fractures, ten fractures resulted in non-unions. 8 of these 10 non-unions were shaft fractures; additionally a collum and a supracondylar non-union were noted (Tables 2, 3; Fig. 1).

**Table 2:** Patient characteristics comparing union and non-union groups in osteogenesis imperfecta, all femur fractures were included if age ≥ 16 years

	Union (*n* = 39)	Non-union (*n* = 10)
Age (years)	44.9 ±14.4	45.5 ±13.1
Age at fracture (years)	36.3 ± 13	40.1 ± 13.04
Gender (male,%)	18 (46.2)	3 (30)
OI type (*n*, %)		
1	19 (48.7)	3 (30)
2	0 (0)	0 (0)
3	12 (30.7)	1 (10)
4	7 (18)	6 (60)
5	1 (2.6)	0 (0)
Type of fracture (*n*, %)		
Proximal	11 (28.2)	1 (10)
Shaft	17 (43.6)	8 (80)
Distal	11 (28.2)	1 (10)
Type of treatment (*n*, %)		
Plate fixation	9 (23.1)	2 (20)
Intramedullary fixation	13 (33.3)	2 (20)
Conservative	15 (38.5)	6 (60)
Unknown	2 (5.1)	

Data are presented as mean ± standard deviation or number of patients and percentages of group

**Table 3:** Patient characteristics of patients with non-union

Patient	Gender (M/F)	Age	Age at fracture	Total of femur fractures	Type 01	Type fracture	Side (L/R)	Treatment
1^a^	M	43	32	2	4	Shaft	R	Conservative
2^a^	M	43	38	2	4	Shaft	R	Conservative
3	F	54	45	2	4	Proximal	L	Conservative
4	F	59	**–**	1	4	Shaft	L	Conservative
5	M	21	20	2	4	Shaft	L	Conservative
6	F	25	20	1	1	Shaft	L	Conservative
7	F	60	56	2	4	Shaft	L	Plate fixation
8	F	51	48	1	1	Distal	L	Plate fixation
9	F	51	50	1	1	Shaft	L	Intramedullary fixation
10^b^	F	49	47	2	3	Shaft	L	Intramedullary fixation

*F* female, *M* male, *L* left, *R* right

^a^Same patient

^b^Died; death was not related to fracture

### Influence of treatment on healing rate

**Fig. 2:**
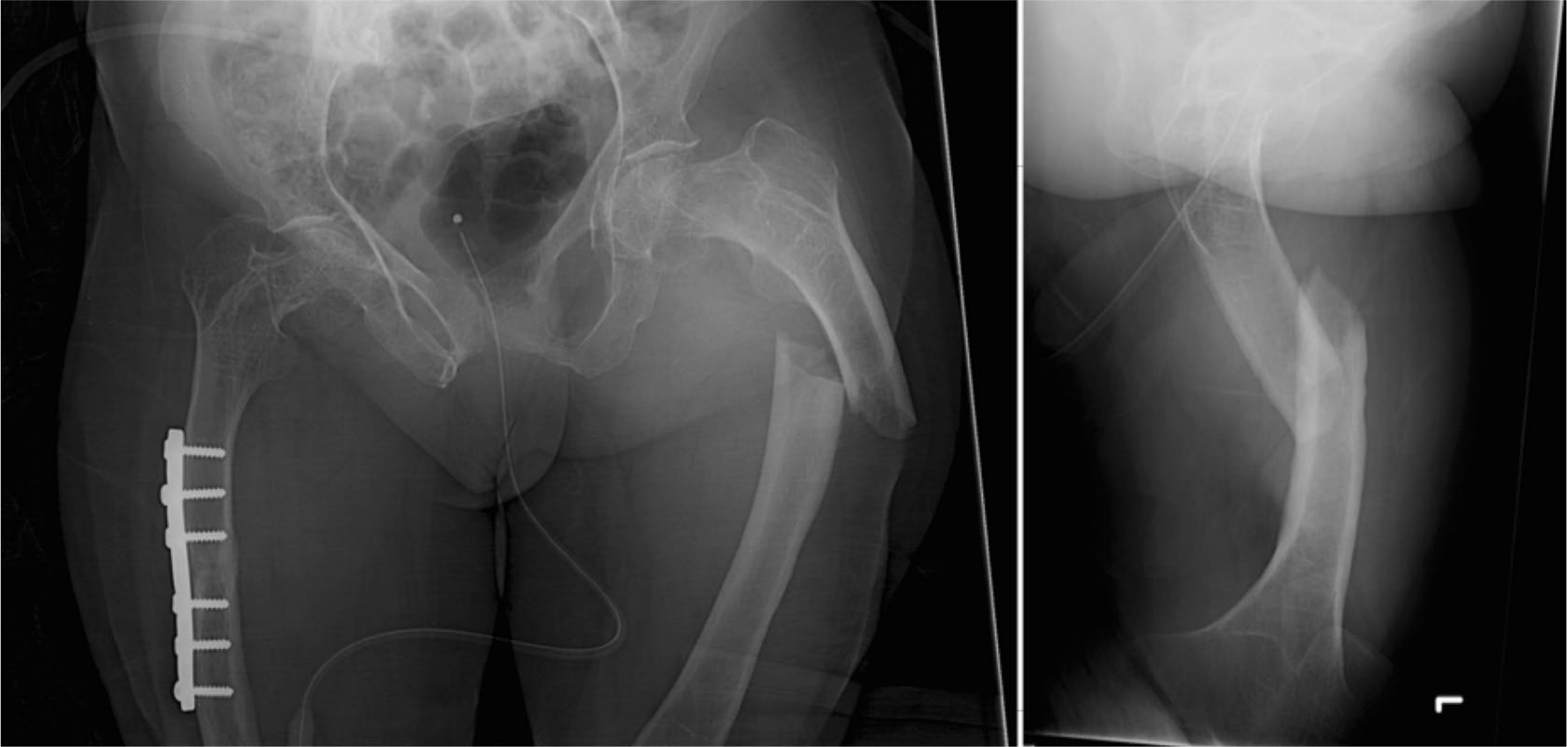
A 60-year-old type 4 OI female (patient number 7 in Table 3) suffered a left midshaft femur fracture in February 2011

**Fig. 3:**
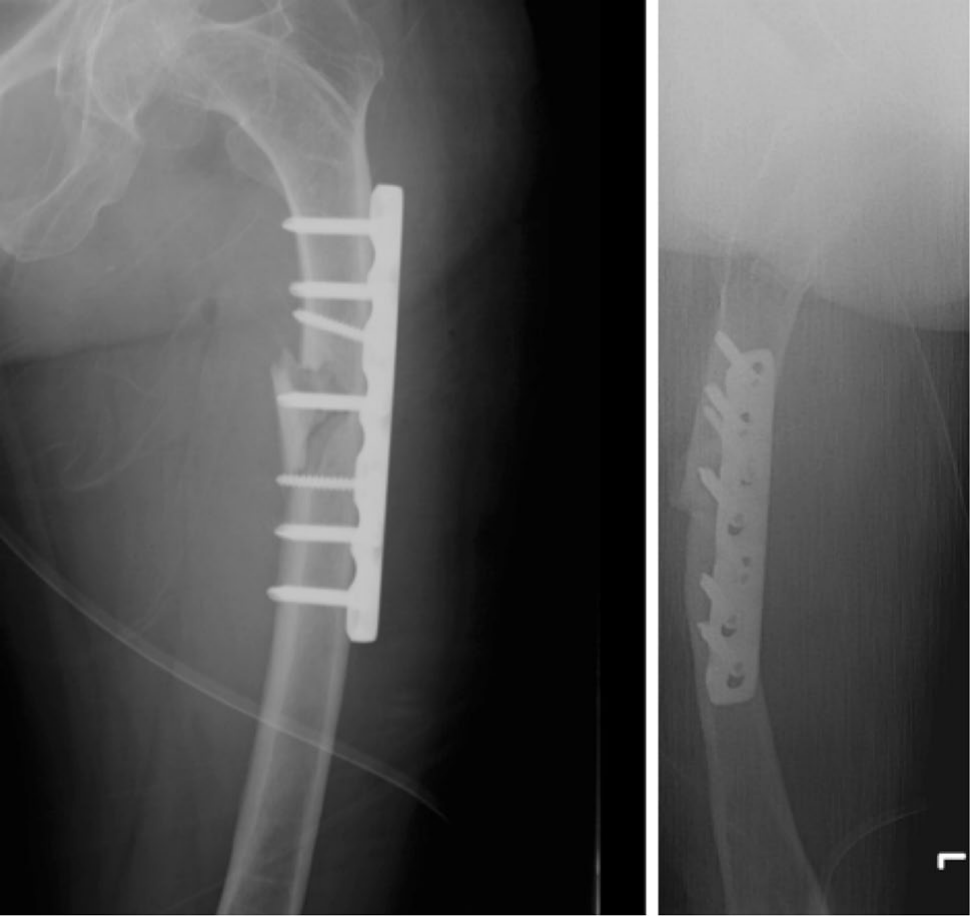
An open reduction internal fixation using a Locking Compression Plate (LCP 7 holes) was performed in February 2011

**Figs. 4–5:**
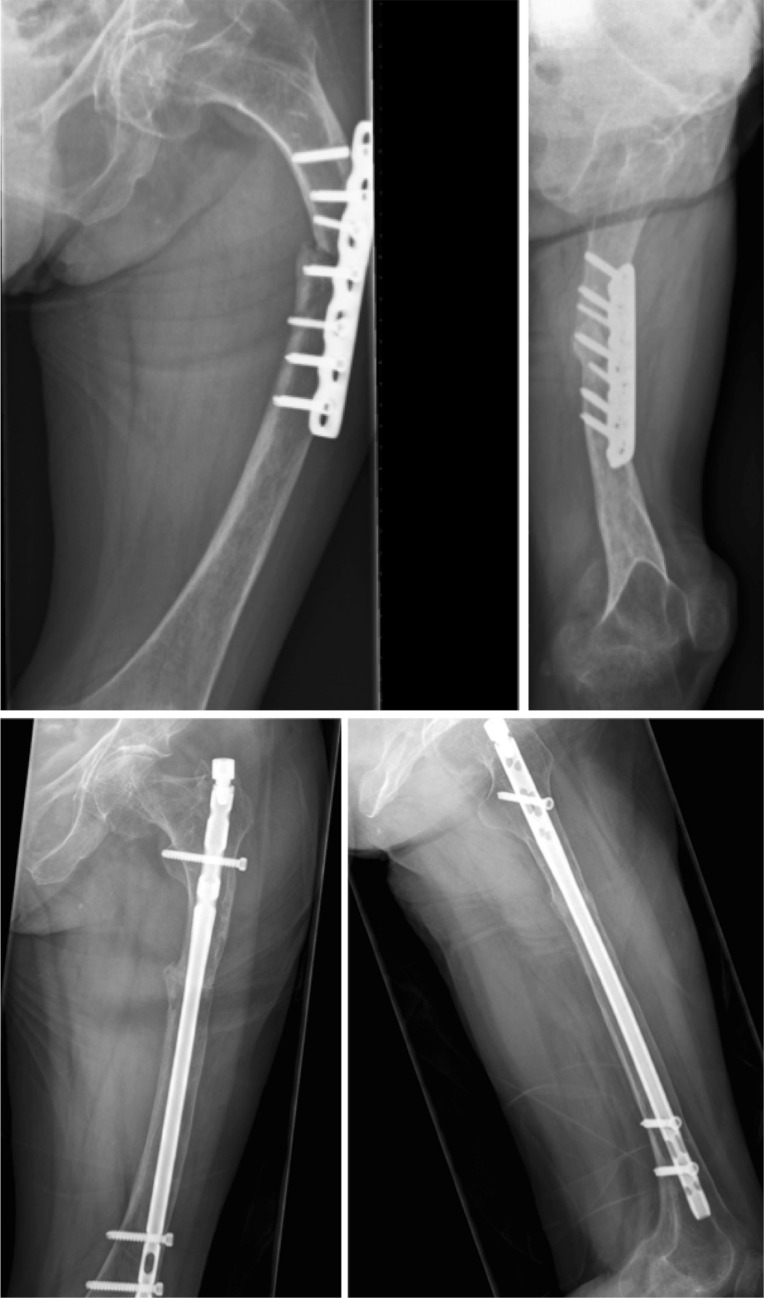
Follow-up in September 2012 showed pseudoarthrosis. Pseudoarthrosis repair was performed in November 2012 using an intramedullary femoral nail (T2) combined with wedge excision for correcting varus deformity. The nail was mobilized in April 2013, resulting in union

In OI type 4, 6 out of 13 (46.2%) fractures resulted in non-union. All four conservatively treated type 4 OI patients having shaft fractures resulted in non-unions. Four other type 4 patients were surgically treated for shaft fractures. Three resulted in union (2 IN, 1 PF); one plate-fixated fracture resulted in non-union (Fig. 2, 3, 4, 5). The sixth type 4 OI patient, having a non-union, had a conservatively treated collum fracture.

In OI patients with type 1 OI, 3 out of 22 (13.6%) fractures resulted in non-union, for type 3, 1 out of 13 (7.7%) fractures resulted in non-union. 1 out of only 2 known type 5 OI patients had a femur fracture. This conservatively treated supracondylar fracture resulted in union.

Overviewing all shaft fractures (*n* = 25), intramedullary fixated fractures resulted in two non-unions out of nine fractures (22.2%). Plate-fixated fractures resulted in one non-union out of five fractures (20%). Conservatively treated shaft fractures resulted in five non-unions out of nine fractures (55%). Two other patients suffered shaft fractures, however, data on type of treatment and outcome are unknown.

## Discussion

In this study, we reviewed 216 adult OI patients on the incidence of femur fractures and non-unions, presenting a total of 49 femur fractures (22.7%) and 10 non-unions (20.4%). Especially conservatively treated midshaft fractures in type 4 OI adults were prone to developing non-unions. For all types of femur fractures, the incidence was 651 fractures per 100,000 person-years annually. For shaft fractures, the incidence was 355 fractures per 100,000 person-years annually. These results reveal a convincing discrepancy when compared to non-OI patients, where its incidence for adult femoral shaft fractures is around 10 per 100,000 person-years annually [14, 15].

This study is the first in describing the incidence and non-union rate of femur fractures in OI adults, presumably explained by the rarity of OI. Some smaller studies compared outcomes of different treatment options in children with OI. Chiarello et al. retrospectively compared surgical versus conservative treatment of 29 children with long bone fragility fractures, reporting a slightly lower non-union and delayed-union rate when treated surgically [16]. Enright and Noonan described bone plating of femur and tibia fractures in four children with OI type 3, resulting in high complication rates [17]. Agarwal and Joseph and Gamble et al. found a 15–20% prevalence on fracture non-union in a heterogeneous group of OI children over a 10–14 year period [18, 19]. However, these number of subjects are small and the results could presumably not be extrapolated to adults, whereas non-OI adults have a 7.3 times higher non-union rate compared to non-OI children [20].

### Patient-dependent factors

As our study reveals new insights on incidence of femur fractures and non-union in OI adults, it has notifiable limitations when analyzing for non-union. Effort was taken to integrate causative factors for non-union, which included bisphosphonate use, DXA scans, smoking status, nutritional deficiency, vitamin D deficiency, mobilization status, metabolic disease or endocrine pathology [21]. We were not able to adjust for these confounders, as information was often not available due to lost data.

### Recommendations for shaft fractures

Although this study has its limitations, we could state that conservative treatment in femoral shaft fractures in OI adults has a high tendency for non-unions. Reviewing our data, we recommend a surgical approach in case of shaft fractures. Despite the fact that our study results in no definite consensus on the optimum surgical treatment for femoral shaft fractures, we favor intramedullary nailing. Intramedullary nailing is the standard treatment in case of a femoral shaft fracture in non-OI adults, which provides the advantages of optimal mechanical stability, efficient load transfer, minimization of stress concentration, early mobilization of hip and knee, preservation of soft tissues, fracture hematoma and periosteal blood supply [13, 22–24]. Karadimas et al. conducted a literature review on complications using intramedullary nails in non-OI femoral fractures, reporting a non-union rate of 1–14.1% [25]. Excluding the study of Noumi et al., which only included open fractures, non-union rates dropped to 1–7.6% [26, 27]. In case of anatomical abnormalities, a smaller nail could offer a solution. We often use a humeral nail in case of a narrow intramedullary canal, applying a wedge osteotomy if needed.

Care should be personalized to patients’ characteristics, type of fracture, anatomical situation and pre-existent materials. Centralization and multidisciplinary work-up (orthopaedic surgeon, anesthetist, rehabilitation specialist, physiotherapist, occupational therapist, internist, geneticist, and radiologist) should be endeavored.

In conclusion, the results of this study showed high incidence of femur fractures and non-union rates in adult OI patients. Conservatively treated shaft fractures in type IV patients were prone to developing non-unions. Although results should be interpreted carefully, as confounding factors were not analyzed, this study provides valuable features of a unique collection of OI patients. Larger cohort studies are needed to approve the best treatment options on femur fractures in adult OI patients.
